# Gallbladder and gastric metastasis as initial presentation of an undiagnosed primary lobular breast carcinoma

**DOI:** 10.1093/omcr/omag009

**Published:** 2026-02-24

**Authors:** Blagica Krsteska, Sanja Ivkovska, Vanja Filipovski, Katerina Kubelka Sabit, Dzengis Jasar, Tahir Senol, Magdalena Bogdanovska Todorovska

**Affiliations:** Institute of Pathology, Faculty of Medicine, University Ss. Cyril and Methodius, 50 Divizija 6, Skopje 1000, Republic of North Macedonia; Institute of Pathology, Faculty of Medicine, University Ss. Cyril and Methodius, 50 Divizija 6, Skopje 1000, Republic of North Macedonia; Department of histopathology and cytology, Clinical Hospital Acibadem-Sistina, Skupi 5A, Skopje 1000 North Macedonia; Faculty of Medical Sciences, Goce Delcev University, Krste Misirkov 10-a, Stip 2000, North Macedonia; Department of histopathology and cytology, Clinical Hospital Acibadem-Sistina, Skupi 5A, Skopje 1000 North Macedonia; Faculty of Medical Sciences, Goce Delcev University, Krste Misirkov 10-a, Stip 2000, North Macedonia; Department of histopathology and cytology, Clinical Hospital Acibadem-Sistina, Skupi 5A, Skopje 1000 North Macedonia; Faculty of Medical Sciences, Goce Delcev University, Krste Misirkov 10-a, Stip 2000, North Macedonia; University Surgical Hospital St. Naum Ohridski, 11 Octomvri 53, Skopje 1000, North Macedonia; Institute of Pathology, Faculty of Medicine, University Ss. Cyril and Methodius, 50 Divizija 6, Skopje 1000, Republic of North Macedonia

**Keywords:** lobular breast carcinoma, gallbladder metastasis, gastric metastasis, immunohistochemistry

## Abstract

The most common sites for breast cancer metastases include the bones, lungs, liver, and brain. Metastases in the GI tract are rare and predominantly originate from lobular breast cancer. Gastric involvement by invasive lobular carcinoma (ILC) is rare and can mimic primary gastric malignancies, leading to diagnostic challenges. Metastasis of ILC to the gallbladder is exceedingly rare and often identified incidentally during cholecystectomy performed for presumed benign conditions. We present a case of a 62-year-old female patient with symptoms of weight loss and dysphagia. After CT and gastroscopy, gastrectomy and cholecystectomy were performed due to suspicion of gastric carcinoma. Histology and immunohistochemical profiling, with estrogen receptor (ER), progesterone receptor (PR), E-cadherin, GATA3, Mammaglobin, and GCDFP-15, favored the diagnosis of lobular breast carcinoma metastasis over primary gastric adenocarcinoma. Awareness of these atypical presentations is crucial for accurate diagnosis and effective management, as misdiagnosis can result in suboptimal treatment strategies.

## Introduction

According to GLOBOCAN 2022 data, breast cancer is the most common cancer in women, with an estimated 2.3 million new cases and 666 000 deaths globally. The advanced stages of breast cancer advanced onset of mortality. The most common sites for breast cancer metastases include the bones, lungs, liver, and brain. Metastases in the GI tract are rare and predominantly originate from lobular breast cancer. Invasive lobular carcinoma is a distinct subtype of breast carcinoma, occurring in 4%–18% of breast cancer cases. Although invasive ductal carcinoma is more common, lobular carcinoma has been found to spread to the GI tract more often. The time interval between breast cancer and the presence of metastases to the GI tract is thought to be approximately from 3 months to 30 years, with a more prevalent occurrence 4–5 years after the initial diagnosis [1].

**Figure 1:**
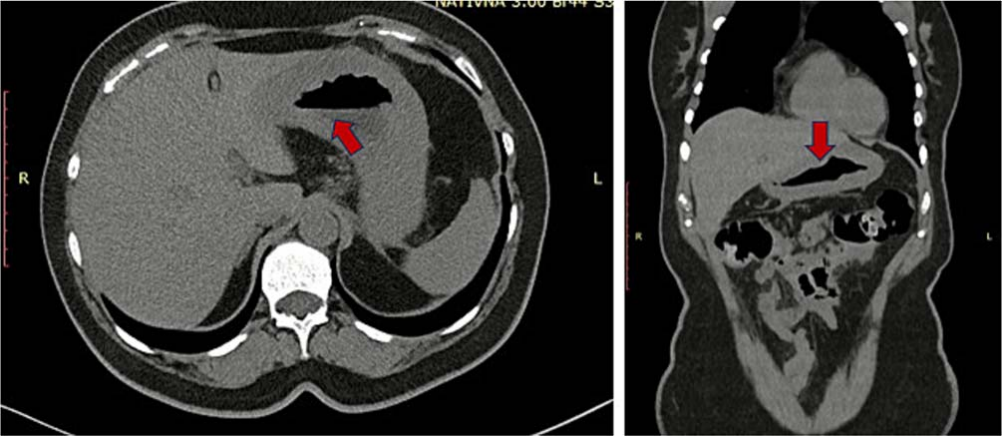
CT scan before surgery (red arrow indicating thick gastric wall).

**Figure 2:**
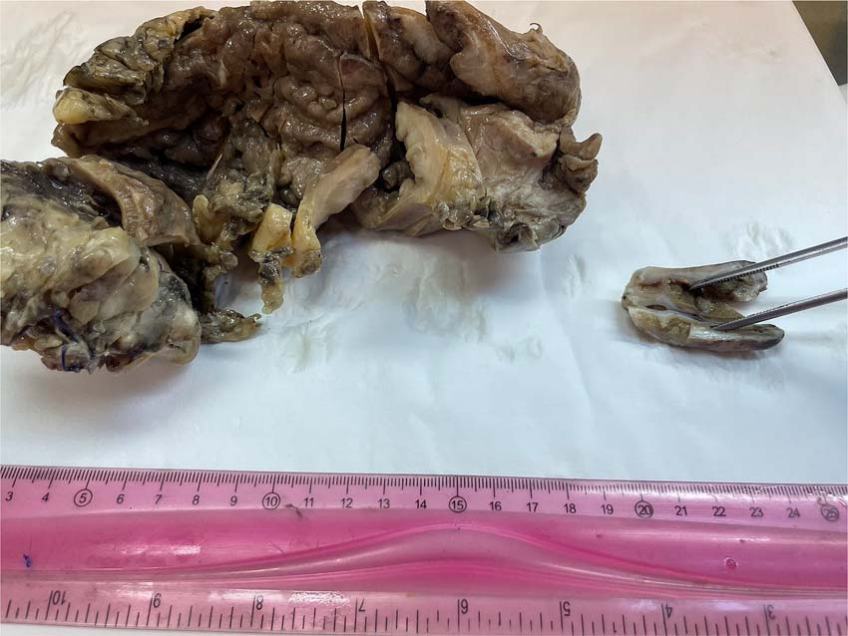
Gross surgical specimens from gastrectomy and cholecystectomy.

Invasive lobular carcinoma disseminates within the breast parenchyma through diffuse infiltration, typically characterized by single-file arrangements of malignant cells. This pattern results in minimal architectural disruption and elicits a limited stromal response. The loss of E-cadherin expression, a key cell–cell adhesion molecule, in invasive lobular carcinoma has been proposed as a contributing factor to its distinct metastatic pattern compared to that of invasive ductal carcinoma. The most common sites of metastases of lobular carcinoma were the bone (81%), followed by the gastrointestinal tract (32%), liver, the gynecological organs, and peritoneum/retroperitoneum [2].

Metastases to the GI tract may sometimes be the initial manifestation of undiagnosed breast cancer, usually involving the stomach (6%–18%) and presenting as ‘linitis plastica’ due to the diffuse intramural infiltration of the stomach, with narrowing of the stomach’s lumen, rigidity and diminished peristalsis, followed by the colon and rectum (8%–12%), while metastasis in the small bowel seldom occurs. Metastatic involvement of the lower gastrointestinal tract is uncommon and frequently presents as multifocal lesions. Gastrointestinal involvement may occasionally manifest with clinical complications such as bowel obstruction, hemorrhage, or perforation. Peritoneal metastases typically appear as small nodules, which can coalesce over time. Retroperitoneal dissemination may lead to ureteric obstruction and subsequent hydronephrosis [3].

**Figure 3:**
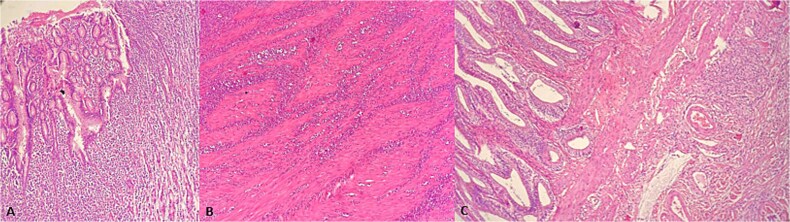
HE x100. (A) Gastric mucosa infiltration with diffuse tumor cells. (B) Gastric muscular wall with diffuse tumor infiltration. (C) Gallbladder wall with diffuse tumor infiltration.

**Figure 4:**
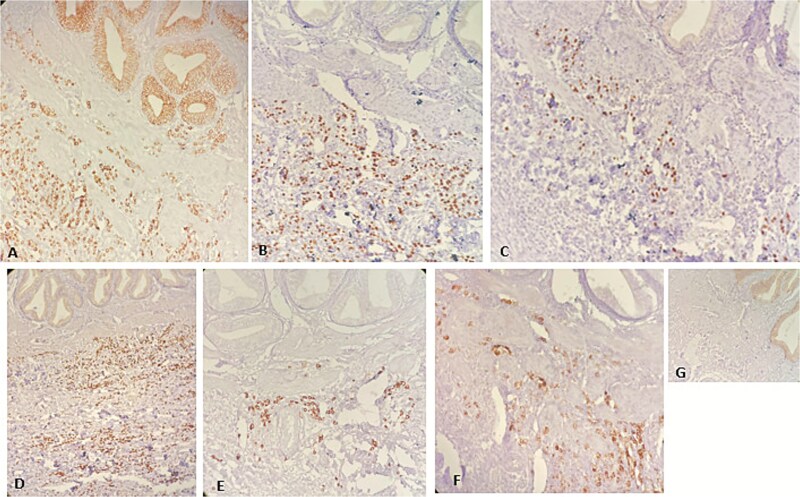
Immunohistochemistry. (A)CK7x100. (B) ERx200. (C) PRx200. (D)GATA3x100 (E)Mammaglobinx200. (F)GCDFP-15x200. (G) E-cadherinx200.

## Case report

A 62-year-old female patient with recurrent urinary infection and long-term leucopenia was admitted to the hospital for dysphagia and a weight loss of 6 kg in two months. She had no family history of cancer. An enhanced CT of the abdomen revealed a thickened gastric wall with a contracted gallbladder, with normal liver and pancreas findings (Fig. 1). Gastroscopy showed an infiltrative scirrhous gastric lesion with hard and confluent mucosal folds. The biopsy revealed scant material with only a few poorly cohesive cancer cells, and a diagnosis of gastric adenocarcinoma was made. Total gastrectomy and a cholecystectomy were performed (Fig. 2). There was a gastric infiltrative neoplasm measuring 6x9cm with a thickened wall and, linitis plastica “morphology. The gallbladder measured 5x1cm with wall thickening of 0,6 cm. Histological analysis revealed a diffuse infiltrative neoplasm of poorly cohesive tumor cells in the gastric wall and the wall of the gallbladder (Fig. 3). The tumor cells were sparing the gastric glands and diffusely spread through the muscularis propria to the serosa. Immunohistochemistry showed that tumor cells expressed positive staining for estrogen receptor (ER), progesterone receptor (PR), GATA3, Mammaglobin, GCDFP-15, and loss of E-cadherin (Fig. 4). This immunophenotype supports a breast origin rather than a primary gastric carcinoma, as gastric adenocarcinomas are typically negative for hormone receptors (ER/PR) and GATA3. The presence of mammaglobin and GATA3, both breast lineage markers, further confirms the diagnosis (Table 1). In contrast, primary gastric carcinomas often express CDX2, which was negative in this case, reinforcing the metastatic nature of the lesion.

**Table 1:** Immunohistochemical profile distinguishing metastatic lobular breast carcinoma from primary gastric adenocarcinoma.

Antibody	Metastatic Lobular Breast Carcinoma	Primary Gastric Adenocarcinoma	Diagnostic Significance
ER (Estrogen Receptor)	Positive	Negative or rarely weak	Indicates breast origin
PR (Progesterone Receptor)	Positive	Negative	Supports breast origin
GATA3	Positive	Usually Negative	Sensitive and specific marker for breast differentiation
Mammaglobin	Positive	Negative	Specific for breast carcinoma
CK7	Positive	Variable (often positive)	Common in both, but not discriminative alone
E-cadherin	Often reduced or absent in lobular type	Usually positive	Loss supports lobular carcinoma
CDX2	Negative	Positive	Marker of gastrointestinal differentiation
GCDFP-15	Positive	Negative	Additional marker for breast origin
HER2	Variable	Variable	Overlap possible, not decisive alone

After a revision of unexplained hemoperitoneum, the patient died two weeks after the surgical procedure, at the time of diagnosis, and the primary breast carcinoma was not detected. The cause of death was cardio-respiratory insufficiency.

## Discussion

Invasive lobular carcinoma (ILC) of the breast, while accounting for approximately 10%–15% of invasive breast cancers, exhibits unique metastatic patterns compared to the more common ductal subtype. The defining feature of invasive lobular carcinoma (ILC) is the loss of function of the adherens junction complex, most commonly caused by inactivating mutations of the E-cadherin gene. E-cadherin is a homotypic cell–cell adhesion molecule essential for preserving epithelial cohesion. When its function is lost, tumor cells become dyscohesive and infiltrate the surrounding tissue in the characteristic single-file pattern, establishing ILC as a distinct morphomolecular entity. Recognizing ILC as a separate subtype of breast cancer is important, as it shows clear clinical and biological differences from the more common invasive breast carcinoma of no special type (IBC-NST).

Notably, ILC has a predilection for metastasizing to unusual sites, including the gastrointestinal tract and gallbladder, sometimes presenting as the initial manifestation of the disease.

Gastric involvement by ILC is rare and can mimic primary gastric malignancies, leading to diagnostic challenges. Several case reports have documented instances where patients presented with gastrointestinal symptoms, and subsequent investigations revealed gastric metastases from previously undiagnosed ILC. They presented with gastric outlet obstruction, anemia, or weight loss, presumably by primary gastric malignancy [4–6]. Digestive tract endoscopy typically reveals no distinctive changes, and the morphological differences between this condition and primary poorly differentiated gastric adenocarcinoma under pathological examination are minimal, making misdiagnosis highly likely.

Metastasis of ILC to the gallbladder is exceedingly rare and often identified incidentally during cholecystectomy performed for presumed benign conditions: abdominal colic, cholecystitis, and cholelithiasis [7–10].

These cases underscore the importance of considering metastatic ILC in the differential diagnosis when encountering gastric or gallbladder lesions, especially in female patients, even in the absence of a known breast primary. Immunohistochemical profiling, including markers such as estrogen receptor (ER), progesterone receptor (PR), and E-cadherin, plays a crucial role in differentiating metastatic ILC from primary gastrointestinal or gallbladder malignancies. The standard systemic treatment for metastatic estrogen receptor–positive (ER+) and progesterone receptor–positive (PR+) breast cancer is primarily hormone therapy, often combined with targeted agents. However, in the presented case, this treatment option was not feasible. Awareness of these atypical presentations is crucial for accurate diagnosis and effective management, as misdiagnosis can result in suboptimal treatment strategies. Although rare, metastatic lobular carcinoma should be considered when diagnosing poorly cohesive gastric carcinoma from a biopsy.
